# Conservative treatment results of 39 patients with adolescent idiopathic scoliosis

**DOI:** 10.1186/1748-7161-7-S1-O47

**Published:** 2012-01-27

**Authors:** H Yilmaz, T Kuru

**Affiliations:** 1 Canakkale Onsekiz Mart University PMR Department, Canakkale, Turkey; 2 Istanbul University, School of Physical Therapy and Rehabilitation, Istanbul, Turkey

## Background

Scoliosis conservative treatment’s goal is to maintain function, and prevent symptoms in the short and long-term and protect the health related quality of life. The purpose of this study was to analyze our conservative treatment results.

## Materials and methods

39 patients with AIS who did received a conservative treatment method included in the study allocated into three groups. 35 of our patients were female and 4 were male. 4 patients with 20°≥ Cobb angle enrolled into first group (exercise group). 24 patients did not want to receive an exercise programme so they included in 2. group (brace group). All of the patients worn CAD/CAM system-Chêneau-brace.11 patients in the 3. group worn brace and participated outpatient exercise programme. The mean 3 months changes -Treatment results- of three groups were analysed with SPSS.

## Results

There were significant within-group pre-post treatment improvements of Cobb angle for brace and brace+exercise group. Analyses showed no significant differences in other parameters (see Table 1).

**Table 1:** Treatment results after 3 months

Treatment groups	Variables	Before treatment Mean ± SD	After treatment Mean ± SD	P value
**Exercise and brace group (n:11)**	**Max Cobb°**	34.38±9.24	29.37±10.86	**0.024**
	**Vertebral rotation°**	10.63±5.67	9.57±5.62	0.162
	**Rib Hump°**	15.55±9.70	11.66±8.01	0.120

**Brace group (n:24)**	**Max Cobb°**	34.00±8.56	28.60±10.24	**0.000**
	**Vertebral rotation°**	8.82±4.59	7.21±4.46	0.072
	**Rib Hump°**	24.18±14.62	19.25±12.76	0.328

**Exercise group (n:4)**	**Max Cobb°**	20.56±3.00	19.28±1.00	0.664
	**Vertebral rotation°**	5.50±1.29	4.52±2.12	0.205
	**Rib Hump°**	4.00±6.92	4.26±3.54	0.653

## Conclusion

In the conservative treatment of AIS, making the right decision about patient, brace and exercise is very important.

Our study have some limitations, our follow up time was short and we did not have enough patients in each group so we could not compare groups results.

